# Deep Neck Space Infections: A Study of 76 Cases

**Published:** 2015-07

**Authors:** Gaurav Kataria, Aditi Saxena, Sanjeev Bhagat, Baldev Singh, Manpreet Kaur, Gurpreet Kaur

**Affiliations:** 1 *Department of Otorhinolaryngology, SGRDIMSR, Amritsar, Punjab, India*.; 2 *Department of Pathology, GMC, Amritsar, Punjab, India*.; 3 *Department of Otorhinolaryngology, GMC, Patiala, Punjab, India*.

**Keywords:** Deep neck space infection, Incision and drainage, Ludwig’s angina, Odontogenic infections, Peritonsillar abscess, Submandibular abscess, Tonsillar and pharyngeal infections, Tracheotomy

## Abstract

**Introduction:**

Deep neck space infections (DNSI) are serious diseases that involve several spaces in the neck. The common primary sources of DNSI are dental infections, tonsillar and salivary gland infections, malignancies, and foreign bodies. With widespread use of antibiotics, the prevalence of DNSI has been reduced. Common complications of DNSI include airway obstruction, jugular vein thrombosis, and sepsis. Treatment principally comprises airway management, antibiotic therapy, and surgical intervention. This study was conducted to investigate the age and sex distribution of patients, symptoms, presentation, sites involved, bacteriology, and management and complications of DNSI.

**Materials and Methods:**

This retrospective study was performed from October 2010 to January 2013, and included 76 patients with DNSI. Patients of all age groups and gender were included. All parameters including age, gender, co-morbidities, presentation, site, bacteriology, complications, and required interventions were studied.

**Results:**

In our study, the majority of patients were in the 31–50-year age group. Males accounted for 55.26% of the sample and females for 44.74%, with a male:female ratio of 1.23. Most of the patients were from a rural background. Diabetes was found as a co-morbid condition in 10.52% cases. Neck pain was the most common symptom, identified in 89.47% cases. The most common etiological factor was odontogenic infection (34.21%), followed by tonsillar and pharyngeal infection (27.63%). The most common presentation was Ludwig’s angina (28.94%), followed by peritonsillar abscess and submandibular abscess. In 50% of cases, Streptococcus and Staphylococcus were found in the culture. Surgical intervention was carried out in 89.47% cases. Emergency tracheotomy was required in 5.26% cases.

**Conclusion:**

DNSI can be life-threatening in diabetic patients, the immunocompromised, and elderly patients, and special attention should therefore be given to these groups. Early diagnosis and treatment is essential to prevent complications. All patients must be treated initially with intravenous antibiotics, with treatment subsequently updated based on a culture and sensitivity report. Due to poor oral hygiene, lack of nutrition, smoking and chewing of beetle nut and tobacco, odontogenic infections are the most common cause of DNSI. Thus, DNSI could be prevented by making the population aware of dental and oral hygiene and offering regular check-ups for dental infections.

## Introduction

Deep neck space infection (DNSI) refers to an infection in the potential spaces and fascial planes of the neck, either with abscess formation or cellulitis (1). At least 11 deep spaces are part of the complex structure formed by the facial planes, providing possible infection sites. Based on their relationship with the hyoid bone, the deep spaces may be classified as follows: spaces localized above the hyoid level (peritonsillar, submandibular, parapharyn- geal, buccal, parotid, masticatory/ temporal); spaces that involve the entire circumference of the neck (retropharyngeal, danger space, prevertebral and carotid); and the anterior or pretracheal visceral space, below the hyoid bone (2).

DNSI are bacterial infections originating from the upper aerodigestive tract and involving the deep neck spaces (3). The most common primary sources of DNSI are the dentition, tonsils, salivary glands, foreign bodies, and malignancies. DNSI often occur following preceding infections such as dental caries, tonsillitis, pharyngitis, trauma to the head and neck, or among intravenous drug abusers. Infections originating from teeth or their supporting structures, known as odontogenic infections, are among the most common diseases in the oral and maxillofacial region, especially in developing countries (4). Previously, before the advent of antibiotics, tonsillar and peritonsillar infections were the source of infection in 70% cases of DNSI (5); but now the most common cause is considered to be dental in origin (6). DNSI are usually polymicrobial in nature. Streptococci, Peptostreptococcus species, Staphylococcus aureus, and anaerobes are the most commonly cultured organisms from DNSI (7,8). Clinical manifestations of DNSI depend on the spaces involved, and include pain, fever, malaise, fatigue, swelling, odynophagia, dysphagia, trismus, dysphonia, otalgia, and dyspnea (9). 

A rapidly progressive course of DNSI with a fatal outcome may be seen, especially in immunocompromised patients with diabetes mellitus, chemo- therapy, steroid therapy, or HIV infection (2). In past, these infections were fairly common; however, with the advent of broad spectrum antibiotics, the incidence of these infections has decreased. Despite the extensive use of antibiotics, DNSI still remains serious and is associated with significant morbidity. These infections are potentially life threatening and spread rapidly, leading to severe complications. Potentially life-threatening complications have been reported to occur at a rate of 10–20%, even in recent literature on DNSI cases (10,11). 

Common and potentially life-threatening complications include airway obstruction, jugular vein thrombosis, descending mediastinitis, pericarditis, pleural empyema, cavernous sinus thrombosis, sepsis, respiratory distress, disseminated intra- vascular coagulation (2), pleuropulmonary suppuration, and hematogenous dissemination to distant organs (12). 

Treatment of DNSI includes antibiotic therapy, airway management and surgical intervention. Management of DNSI is traditionally based on prompt surgical drainage of the abscess followed by antibiotics or nonsurgical treatment using appropriate antibiotics in the case of cellulitis (13). Proper diagnosis and prompt management can effectively overcome the disease and provide a cure without complications. However, for this to be possible, otorhinolaryngologists must have detailed knowledge of the presentation, etiology, investigations and access to appropriate medical and surgical interventions. The main aim of our study was to share our experience in terms of presentation, clinical trends, common sites involved, bacteriology, management, complications, and outcomes.

## Materials and Methods

In this retrospective study, 76 patients who were admitted and treated for DNSI in the ear, nose and throat (ENT) department from October 2010 to January 2013 were included. We excluded patients who had an infection related to inhalant injuries or due to any malignancy. A total of 76 cases of DNSI met our inclusion criteria. Patients of all age groups and both genders were included. All parameters including age, gender, co-morbidities, symptoms, site involved, bacteriology, culture growth, type of intervention required, complications, and outcome were studied. All patients were initiated on treatment with amoxicillin, clavulanate, and metronidazole; the treatment regimen was later modified based on a culture and sensitivity report.

## Results

We evaluated 76 DNSI inpatients. The mean age of the patients was 33.2 years, with a minimum age of 4 years and a maximum age of 72 years. The majority of patients were in the 31–40-year age group, followed by the 41–50-year age group.

**Table 1:** Sex distribution of patients (n=76).

Sex	No. of patients (%)
Male	42 (55.26)
Female	34 (44.73)
Total	76 (100)

Out of a total of 76 patients, 42 (55.26%) were male and 34 (44.73%) were female; with a male:female ratio of 1.23 (Table. 1). With respect to demographic distribution, 52 patients (68.42%) were from a rural background and 24 (31.58%) were from an urban background. Twenty-eight patients (36.84%) were tobacco chewers and 16 (21.05%) were tobacco smokers. Out of 76 patients, eight (10.52%) had diabetes mellitus, four (5.26%) were intravenous drug users and two (2.63%) had chronic kidney disease. 

**Table 2:** Symptoms of DNSI patients

Symptom	No. of patients (%)
Neck pain	68 (89.47)
Neck swelling	65 (85.52)
Dysphagia	63 (82.89)
Fever	58 (76.31)
Odynophagia	58 (76.31)
Toothache	28 (36.84)
Airway difficulty	08 (10.52)
Trismus	06 (7.89)
Torticollis	03 (3.94)

Neck pain was the most common symptom, found in 68 patients (89.47%), followed by neck swelling in 65 patients (85.52%), dysphagia in 63 patients (82.89%), fever in 58 patients (76.31%), odynophagia in 58 patients (76.31%), toothache in 28 patients (36.84%), airway difficulty in eight patients (10.52%), trismus in six patients (7.89%), and torticollis in three patients (3.94%) (Table. 2).

**Table 3:** Etiological factors in patients with DNSI

Etiology	No. of patients (%)
Odontogenic	26 (34.21)
Tonsillopharyngitis	21 (27.63)
Infected lymphadenopathy	07 (9.21)
Furunculosis	05 (6.57)
Trauma	04 (5.26)
Thyroglossal cyst	02 (2.63)
Salivary gland infections	02 (2.63)
Foreign body	01 (1.31)
Complicated otitis media	01 (1.31)
Unknown	07 (9.21)
Total	76 (100)

In the majority of cases, the etiological factor was odontogenic in origin (26 patients, 34.21%). The etiological factor was unknown in seven patients (9.21%) (Table. 3).

**Table 4:** Location of abscess in DNSI patients (n=76)

Presentation	No. of patients (%)
Ludwig’s angina	22 (28.94)
Peritonsillar abscess	18 (23.68)
Submandibular abscess	14 (18.42)
Parapharyngeal abscess	08 (10.52)
Retropharyngeal abscess	04 (5.26)
Submental abscess	03 (3.94)
Parotid abscess	02 (2.63)
Anterior triangle neck abscess	02 (2.63)
Masticator abscess	02 (2.63)
Posterior neck abscess	01 (1.31)
Total	76 (100)

Ludwig’s angina was the most common clinical presentation of neck abscess, in 22 patients (28.94%), followed by peritonsillar abscess in 18 patients (23.68%), submandibular abscess in 14 patients (18.42%), parapharyngeal abscess in eight patients (10.52%), retropharyngeal abscess in four patients (5.26%), submental abscess in three patients (3.94%), parotid abscess in two patients (2.63%), anterior triangle neck abscess in two patients (2.63%), masticator abscess in two patients (2.63%) and abscess in the posterior region of the neck in one patient (1.31%) (Table.4). The majority of patients (69,90.78%) underwent a computed tomography (CT) scan, four patients (5.26%) had a neck ultrasound, and no imaging was performed in the remaining three patients (3.94%). Routine investigations were performed in all patients.

Out of 76 patients, 68 (89.47%) underwent intervention consisting of either incision and drainage or needle aspiration or both, and a pus specimen from these patients was sent for culture and sensitivity analysis. Forty-six patients had positive culture results. The most common organism cultured was Streptococcus (19,27.94%), followed by Staphylococcus (15,22.05%), polymicrobials (9,13.23%), Klebsiella (4, 5.88%), Pseudomonas (4, 5.88%), Anaerobes (4, 5.88%), E. coli (1, 1.47%), and Proteus (1,1.47%). No bacterial growth was found in 11 patients (16.17%).

In terms of management, incision and drainage, needle aspiration or both were performed in 68 patients (89.47%). Eight patients (10.53%) were managed by medical treatment alone. All 76 patients were given broad spectrum intravenous antibiotics, which were later updated based on culture and sensitivity report. Forty-four patients (57.89%) required an external approach for incision and drainage. Fifteen patients (19.74%) needed intra-oral aspiration, while nine patients (11.84%) required both approaches. Mastoidectomy was performed in one (1.31%) of the patients. Four patients (5.26%) required emergency tracheotomy for airway management. 

Complications were encountered in a few patients. Four patients had upper airway obstruction, three had septic shock, two had internal jugular vein thrombosis, two had skin necrosis and one had mediastinitis. One patient died during his hospital stay due to an unrelated systemic complication. The mean hospital stay was 4.2 days, with a minimum of 3 days and a maximum of 15 days. 

## Discussion

The widespread use of antibiotics has decreased the incidence of DNSI, but it remains a fairly common problem. In our study, 76 DNSI patients were included and all were admitted to the hospital for treatment. The management and diagnosis of DNSI is still a challenge for otorhinolaryngologists. Evaluation of variables relating to life-threatening diseases is very important. In our study, the majority of patients were seen in their third and fourth decade. This correlates with the studies by Parischar et al. and Meher et al. in which 50% and 60% patients were in the third and fourth decade of life, respectively (6,14). In our study, a male predominance was seen, which is consistent with studies by Sethi et al. Meher et al. and Parischar et al. all of which showed male preponderance (6,14,15). Further, in our study, diabetes was associated with 10.52% patients, which is very low compared with the study of Huang et al which reported 30.3% patients of diabetes mellitus (8). Twenty-eight of our patients were tobacco chewers. This results in poor oral hygiene and is reported to affect the host’s vulnerability to systemic diseases by the formation of subgingival biofilms acting as reservoirs of Gram-negative bacteria, and through the periodontium acting as a reservoir of inflammatory mediators (16). 

Our study is consistent with those of Bakir et al. Meher et al. Sethi et al. and Marioni et al. with pain as the most common symptom followed by swelling, dysphagia/odynophagia, and trismus (10,14,15,17). 

The length of hospital stay as an indicator of severity of infection was related to the following factors: socioeconomic status, blood sugar level, hemoglobin level, oral hygiene and white blood cell counts. In a study by Tschiassny et al. 70% of cases of DNSI were odontogenic in origin (18). In a retrospective study by Parhiscar et al. odontogenic infections were declared as the most common cause of DNSI (43%) (6). Bottin et al. also reported similar results to Parhiscar et al. with 42% of DNSI due to odontogenic origin (6,19). Huang et al. Marioni et al. and Eftekharian et al. reported that odontogenic problems were the most common causative factor for DNSI, in 42%, 38.8% and 49% cases, respectively (8,17,20). Studies by Sethi et al. and Har-El G et al. also showed the major cause of DNSI to be dental in origin (15,21).Thus, our study results are consistent with those of these previous studies. The most common presentation of DNSI in our study was Ludwig’s angina (28.94%), followed by peritonsillar abscess (23.68%), submandibular abscess (18.42%), parapharyngeal abscess (10.52%), and retropharyngeal abscess (5.26%). Ludwig’s angina, peritonsillar abscess, and anterior neck abscess accounted for approximately 56% of our cases, which is consistent with the studies by Afshin et al. and Khode et al. with about 60% cases with a similar presentation (22,23). Peritonsillar abscess and submandibular abscess were the second and third most common presentation in our study, which correlates with study results from Pariscar et al. and Stalfor et al. with peritonsillar and submandibular as the second and third most common presentation (6,24).

Streptococcus species were the most common cultured organism in our study, which is consistent with the studies of Ridder et al. Parischar et al. Mumtaz et al. and Gidley et al. (6,25-27). In 11 patients (16.17%), no organism was cultured, which was probably due to use of antibiotics at the time the cultures were sent.

A contrast CT scan is the most appropriate imaging tool, not only for the diagnosis of deep neck space infections, but also to show the extent of disease. CT scans are not only beneficial in differentiating between cellulitis and abscesses, but also have an important role in the evaluation of serious complications. A CT scan also helps to decide whether a surgical intervention is indicated, as patients with radiological evidence of cellulitis respond well to medical treatment, whereas those with abscess have a higher incidence of complications and usually require surgical management due to the aggressive nature of this condition. Ultrasound also plays an important role in the detection of abscess formation (13,28). In our study, a CT scan was performed in 90.78% patients and ultrasound was performed in 5.26% cases.

Worldwide, management of DNSI usually involves early surgical drainage of purulent abscesses via an external incision (1,19,20). In our study, all patients were initiated on intravenous antibiotic therapy with amoxicillin, clavulanic acid, and metronidazole, which was later modified according to the culture and sensitivity report. In the case of a significant abscess on the CT scan, prompt open surgical drainage has been shown to be the most suitable technique for treating DNSI (8). In our study, surgical intervention was carried out in 89.47% of patients, which is consistent with the studies by Mumtaz et al. Eftekharian et al. Parhiscar et al. and Har-El et al. with surgical intervention required in approximately 78%, 79%, 100% and 90% of cases, respectively (6,20,21,26).

Airway management is challenging in patients with DNSI. Usual causes of airway compromise are laryngeal edema and pushing of the tongue upwards and backwards, especially in Ludwig’s angina. In our study, tracheotomy was performed in 5.26% of cases, which is consistent with the study by Eftekharian et al. (20), in which tracheotomy was required in 8.8% cases. A tracheal intubation with rigid laryngoscopy may be difficult in these patients due to the possibility of distortion in the airway anatomy, tissue rigidity, and a limited access to the mouth. Thus, the tracheotomy must always be considered whenever there is respiratory difficulty. Sometimes attempting intubations can worsen an already damaged airway (29). 

## Conclusion

DNSI remains a common and challenging disease for clinicians, and should be treated on emergency basis. It is also very important to give special attention to high-risk groups such as diabetics, the elderly, and patients with underlying systemic diseases as the condition may progress to life-threatening complications. Early diagnosis and treatment is essential. Thus, all patients should be initiated on treatment with empirical intravenous antibiotic therapy, which should be updated later according to the culture and sensitivity report. All patients with a significant abscess on the CT scan require surgical intervention. Tracheotomy should be considered if airway protection is needed. In developing countries, lack of adequate nutrition, poor oral hygiene, tobacco chewing, smoking and beetle nut chewing has led to an increased prevalence of dental and periodontal diseases. In our study, odontogenic infections were the most common etiology for DNSI. Therefore, prevention of DNSI can be achieved by making the population aware of dental and oral hygiene and encouraging regular check-ups for dental infections. 
